# Expression of Nicotinamide Phosphoribosyltransferase-Influenced Genes Predicts Recurrence-Free Survival in Lung and Breast Cancers

**DOI:** 10.1038/srep06107

**Published:** 2014-08-22

**Authors:** Tong Zhou, Ting Wang, Joe G. N. Garcia

**Affiliations:** 1 Arizona Respiratory Center and Department of Medicine, The University of Arizona, Tucson, Arizona, USA; 2 These authors contributed equally to this work.

## Abstract

Nicotinamide phosphoribosyltransferase (*NAMPT*) is a rate-limiting enzyme in the salvage pathway of nicotinamide adenine dinucleotide biosynthesis. NAMPT protein is a secreted plasma biomarker in inflammation and in cancer. The NAMPT enzymatic inhibitor, FK866, acts as an inducer of apoptosis and is a cancer therapeutic candidate, however, little is known regarding the influence of *NAMPT* on cancer biological mechanisms or on the prognosis of human cancers. We interrogated known microarray data sets to define *NAMPT* knockdown-influenced gene expression to demonstrate that reduced *NAMPT* expression strongly dysregulates cancer biology signaling pathways. Comparisons of gene expression datasets of four cancer types generated a N39 molecular signature exhibiting consistent dysregulated expression in multiple cancer tissues. The N39 signature provides a significant and independent prognostic tool of human recurrence-free survival in lung and breast cancers. Despite the absence of clear elucidation of molecular mechanisms, this study validates *NAMPT* as a novel “oncogene” with a central role in carcinogenesis. Furthermore, the N39 signature provides a potentially useful tool for prediction of recurrence-free survival in lung and breast cancer and validates *NAMPT* as a novel and effective therapeutic target in cancer.

Nicotinamide phosphoribosyltransferase (*NAMPT*), also known as pre-B cell colony-enhancing factor 1, encodes a protein that catalyzes the condensation of nicotinamide with 5-phosphoribosyl-1-pyrophosphate to yield nicotinamide mononucleotide, and is the rate-limiting enzyme in the salvage pathway of nicotinamide adenine dinucleotide (NAD) synthesis^1^. This multifunctional enzyme was first cloned from human lymphocytes, and named pre-B cell colony-enhancing factor as a secreted cytokine^2^. *NAMPT* was further confirmed as a pro-inflammatory cytokine^3^ that inhibits neutrophil apoptosis^4^ and exerts endotoxin-like responses to trigger NFκB signaling pathways^5^. Given the dual intracellular enzymatic activity (*iNAMPT*) and extracellular proinflammatory cytokine characteristics (*eNAMPT*), *NAMPT* has been implicated in many important biological processes, including metabolism, stress response, apoptosis and aging^1^, the majority of which are closely related to carcinogenesis signaling.

Secreted *NAMPT* or *eNAMPT* is elevated in plasma in a variety of human cancer types including gastric, endometrial, hepatocellular, colorectal, and breast cancers^6^, indicating circulating *eNAMPT* protein as a potential plasma biomarker or prognosis factor of cancer^7^. Although the molecular mechanism of *eNAMPT* on carcinogenesis is unknown, the inflammatory response of *eNAMPT* on tissues promotes tumor proliferation and redox adaptive responses^8^. Moreover, as an enzyme, intracellular *NAMPT* or *iNAMPT* is responsible for regeneration of intracellular NAD^+^, which is a multifunctional co-factor in many cellular events, such as transcriptional regulation, longevity and caloric-restriction responses, cell cycle progression, apoptosis, DNA repair, circadian rhythms, chromatin dynamics regulation, and telomerase activity, processes closely related to cancer pathogenesis^6^. Thus, inhibition of *NAMPT* enzymatic activity by the highly specific, noncompetitive inhibitor, FK866, induced apoptosis in murine neutrophils^9^ and human liver carcinoma cells^10^. Although potentially mediated by NAD^+^ depletion, the clear mechanism of FK866-mediated apoptosis was not characterized. Additional studies determined that FK-866 produced premature senescence, possibly linked to decreased activity of the NAD^+^-dependent enzyme, SIRT1^11^. Similarly, little is known of the molecular mechanisms by which *iNAMPT* may contribute to oncogenesis or influence human cancer prognosis.

We conducted meta-analysis of genome-wide expression data to identify *NAMPT*-influenced genes implicated in cancer pathobiology. First, we identified differentially expressed genes utilizing microarray data from two independent human cell lines (primary and cancer cells) and wild-type (WT) cells and *NAMPT* knock down (KD) cells. These differentially-expressed genes were denoted as *NAMPT*-influenced genes with gene ontology analysis indicating enriched cancer-related pathways. Second, a prognostic gene signature derived from the *NAMPT*-influenced genes was developed and expression was compared in normal and colon, lung, pancreatic, and thyroid cancers. Thirty-nine *NAMPT*-influenced genes were identified as being commonly differentially expressed in tumor tissues and comprised a multi-molecular cancer outcome predictor. Our studies indicate this molecular signature effectively predicts recurrence-free survival in lung and breast cancer in a manner independent of standard clinical and pathological prognostic factors.

## Results

### *NAMPT*-influenced genes

**Figure 1:**
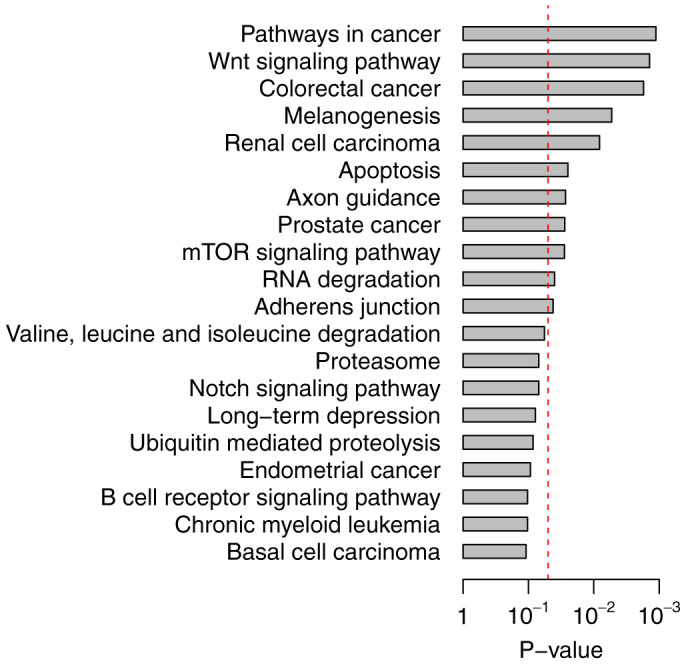
The top 20 KEGG pathway terms associated with the *NAMPT*-influenced genes. The *P*-values were calculated by Fisher's exact test. The red dash line denotes the significance level of *α* = 0.05.

We compared the gene expression pattern between wild type and *NAMPT*-silenced human endothelial cell and a breast cancer cell line to identify genes potentially regulated by *NAMPT*. Two independent microarray datasets containing gene expression information for both wild type and *NAMPT*-silenced cells were collected from the Gene Expression Omnibus (GEO) database^12^: one dataset was derived from a MCF-7 breast cancer cell line (GSE13449)^13^ and the second dataset was from human pulmonary microvascular endothelial cells (GSE34512)^14^. The genes differentially expressed between WT and *NAMPT* silenced cells in both datasets with accordant direction were retained as *NAMPT*-influenced genes. At the specified significance level of false discovery rate (FDR) < 5% and fold change >1.1 (see Methods for details), 462 genes were found to be commonly differentially expressed between WT and *NAMPT* silenced cells, among which 361 genes were up-regulated while 101 genes were down-regulated in *NAMPT* silenced cells (Supplementary Table S1). We next searched the enriched Kyoto Encyclopedia of Genes and Genomes (KEGG)^15^ physiological pathways among the dysregulated genes revealing genes enriched in cancer-related KEGG terms, such as “Pathways in cancer”, “Colorectal cancer”, “Melanogenesis”, “Renal cell carcinoma”, and “Apoptosis” (Figure 1, Fisher's exact test). These findings suggested that the *NAMPT*-influenced genes are involved in human cancer pathology.

**Figure 2:**
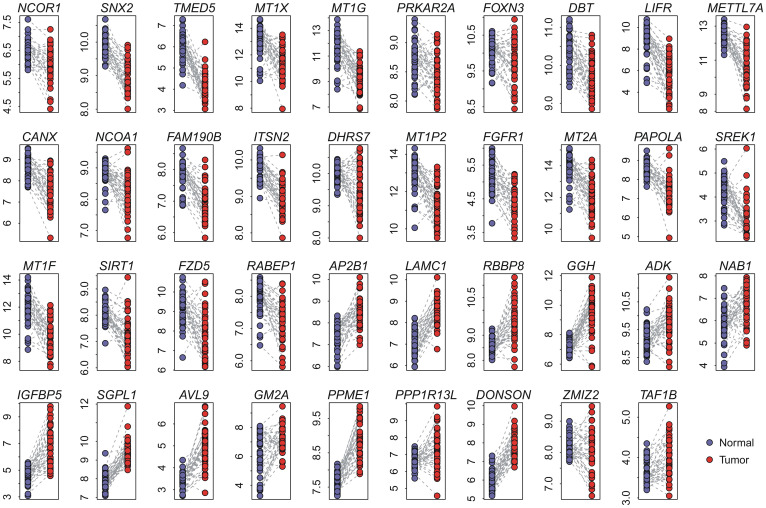
Comparison of N39 gene expression between normal and lung cancer tissues. Paired normal and tumor tissues from 44 lung cancer patients were included in the comparison. All the listed genes are differentially expressed between normal and tumor tissues except *ZMIZ2*. Y-axis: log_2_-transformed expression values.

To determine the depth of involvement of *NAMPT*-influenced genes in human cancers, we explored expression differences of these genes between normal and tumor tissues from lung (GSE18842)^16^, colon (GSE23878)^17^, pancreatic (GSE15471)^18^, and thyroid (GSE33630) cancers. Paired normal and tumor tissues from 44 lung, 19 colon, 36 pancreatic, and 44 thyroid cancer patients were included. Paired t-test was used to detect the differentially-expressed genes between the normal and tumor tissues. In total, 39 genes were identified as mutually differentially expressed and concordant in expression with the *NAMPT*-silenced model (*P* < 0.05 after Benjamini-Hochberg adjustment) in at least three out of four cancer types: lung cancer (Fig. 2), colon cancer (Supplementary Fig. S1), pancreatic cancer (Supplementary Fig. S2), and thyroid cancer (Supplementary Fig. S3). We designated this *NAMPT*-influenced 39-gene set as the N39 gene signature (Table 1).

### N39 predicts recurrence-free survival in lung and breast cancers

We hypothesized that the N39 signature would be predictive of tumor outcome in lung and breast cancer patients. We constructed a scoring system to assign each patient a risk score, representing a linear combination of the N39 gene expression values weighted by the coefficients obtained from training cohorts (GSE8894^19^ for lung cancer and GSE2034^20^ for breast cancer) (see Methods for details). N39-positive patients were defined as those having risk scores greater than the group median. As expected, there was a significantly reduced recurrence-free survival for N39-positive patients in the training cohorts (Supplementary Figure S4 and Table 2).

**Figure 3:**
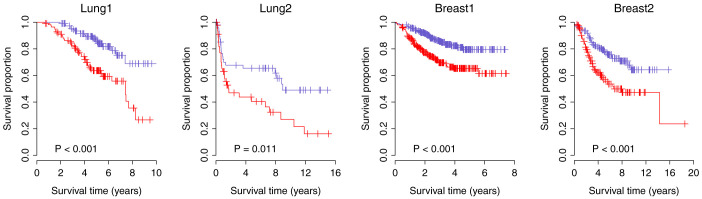
Kaplan-Meier curves for patients in validation cohorts. The expression of N39 predicts poor recurrence-free survival in lung (Lung1 and Lung2 cohorts) and breast (Breast1 and Breast2 cohorts) cancers. Red curves are for N39-positive patients while blue curves are for N39-negative patients. N39-positive patients were defined as those having a risk score greater than the group median. *P*-values were calculated by log-rank tests for the differences in survival between N39-positive and -negative groups.

We tested the ability of the N39 based risk score to classify patients into prognostic groups in independent validation cohorts. For each cancer type, two validation cohorts were collected: Lung1 (GSE31210)^21^ and Lung2 (GSE37745)^22^ for lung cancer, and Breast1 (GSE25066)^23^ and Breast2 (GSE21653)^24^ for breast cancer. Kaplan-Meier survival curves demonstrated a significantly reduced recurrence-free survival for N39-positive patients in the validation cohorts (log-rank test: *P* = 5.4 × 10^−5^ for Lung1; *P* = 0.011 for Lung2; *P* = 2.9 × 10^−5^ for Breast1; and *P* = 7.2 × 10^−4^ for Breast2) (Figure 3). Univariate Cox proportional hazards regression indicated that N39-positive patients exhibited significantly increased risk for recurrence (fold increase or FI) in these 4 cohorts: 2.88-FI for Lung1, 2.08-FI for Lung2, 2.27-FI for Breast1, and 2.12-FI for Breast2 (Table 2). These findings collectively indicate that N39 is predictive of recurrence-free survival in lung and breast cancer.

**Figure 4:**
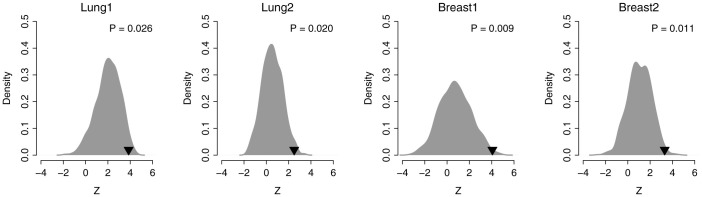
Non-random prognostic power of N39 in lung and breast cancers. *Z* denotes the Wald statistic, the ratio of Cox regression coefficient to its standard error. The black triangles stand for the *Z* values of N39. The grey areas show the distributions of *Z* values for the 1,000 resampled gene signatures with identical size as N39 under the null hypothesis of no association between N39 and recurrence-free survival. Right-tailed *P*-values of the sampling distribution were calculated.

In a recent computational study, 47 published breast cancer prognostic signatures were compared with signatures comprised of randomly selected genes. Approximately 60% of the published signatures were not significantly improved over random signatures of identical size with the majority of random gene signatures significantly associated with breast cancer outcome^25^. We performed a resampling test to determine whether the prognostic power of N39 was significantly better than random gene signatures. We constructed 1,000 random gene signatures of identical size as N39 (39 genes) with Cox proportional hazards regression of survival conducted for each resampled gene signature. The association between each random gene signature and recurrence-free survival was measured by the Wald statistic, the ratio of Cox regression coefficient to its standard error. Our alternative hypothesis was that the Wald statistic value of N39 should be higher than that of randomized gene signatures if N39 was more predictive than randomized signatures. Figure 4 indicates that the Wald statistic of N39 was significantly higher than that of randomized gene signatures (Right-tailed: *P* = 0.026 for Lung1; *P* = 0.020 for Lung2; *P* = 0.009 for Breast1; and *P* = 0.011 for Breast2), suggesting that the null hypothesis that the association between N39 and recurrence-free survival is by chance is rejected.

### N39 is independent of standard clinical and pathological prognostic factors

We investigated the performance of the N39 signature in comparison with standard clinical and pathological factors associated with prognosis in human cancers. For the Lung1 cohort, we considered factors including patient age, gender, smoking history, stage, *EGFR*/*KRAS*/*ALK* gene alteration status, and MYC protein levels. For the Lung2 cohort, we considered age, gender, and stage into account. For the Breast1 cohort, we included factors such as age, lymph node status, histological grade, tumor size, estrogen receptor (ER) status, and progesterone receptor (PR) status. For the Breast2 cohort, factors such as age, grade, ER and PR status, and *TP53* gene alteration status were included in the multivariate model. A multivariate Cox proportional hazards regression of survival indicated that N39 status remained a significant covariate in relation to the clinico-pathological factors in each validation cohorts (*P* = 1.2 × 10^−3^ for Lung1; *P* = 6.4 × 10^−3^ for Lung2; *P* = 2.9 × 10^−3^ for Breast1; and *P* = 1.7 × 10^−2^ for Breast2) (Table 3).

**Figure 5:**
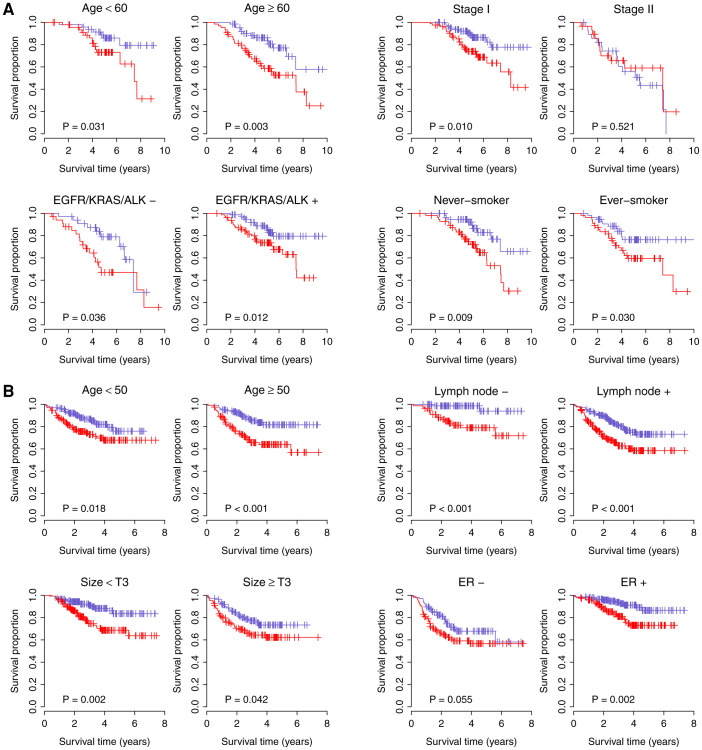
Kaplan-Meier curves of patient cohorts grouped by clinical and pathological factors. (A) N39 is independent of traditional clinicopathological factors in lung cancer. Patients are stratified by age, stage, *EGFR*/*KRAS*/*ALK* alteration status, and smoking history. (B) N39 is independent of traditional clinicopathological factors in breast cancer. Patients are stratified by age, lymph node status, tumor size, and ER status. Red curves are for N39-positive patients while blue curves are for N39-negative patients. N39-positive patients were defined as those having a risk score greater than the group median. *P*-values were calculated by log-rank tests for the differences in survival between N39-positive and -negative groups.

In the Lung2 and Breast2 cohorts, N39 status was the only significant covariate in the multivariate model (Table 3). However, in the Lung1 cohort, patient age, stage, and *EGFR*/*KRAS*/*ALK* alteration status were also significant variables. Therefore, we further stratified the patients in the Lung1 cohort according to respective significant factors and redid Cox proportional hazards regression. For patients aged < 60 and ≥60, N39-positive patients had significant increased risk for recurrence, 2.62-FI (*P* = 0.038) and 2.57-FI (*P* = 0.005), respectively. For patients with stage I cancer (Lung1 cohort only includes patients with stage I and II lung cancer), N39-positive patients exhibited significantly increased risk for recurrence (2.48-FI, *P* = 0.012), however, no significant difference was observed between N39-positive and -negative groups for patients with stage II lung cancer. For patients without and with *EGFR*/*KRAS*/*ALK* alteration, N39-positive patients had a 2.35-FI (*P* = 0.041) and 2.36-FI (*P* = 0.015) increased risk for recurrence, respectively. We also checked the performance of the N39 signature in patients without and with smoking history respectively and found N39-positive patients exhibited increased risk for recurrence in never-smokers and ever-smokers, 2.72-FI (*P* = 0.012) and 2.19-FI (*P* = 0.034) respectively. Kaplan-Meier survival curves demonstrated significantly reduced survival for N39-positive patients in each subset grouped by age, stage, *EGFR*/*KRAS*/*ALK* alteration status, and smoking history, with the exception of patients with stage II lung cancer (Fig. 5A), presumably reflecting the reduced sample size.

In the Breast1 cohort, lymph node status, tumor size, and ER status were significant clinicopathological factors in addition to N39 status (Table 3). We stratified patients in the Breast1 cohort according to these factors. For patients with and without lymph node involvement, N39-positive patients exhibited significantly increased risk for recurrence, 8.03-FI (*P* = 0.006) and 2.09-FI (*P* = 6.1 × 10^−4^), respectively. For patients with tumor size <T3 and ≥T3, N39-positive patients displayed significant increased risk for recurrence, 2.56-FI (*P* = 0.002) and 1.69-FI (*P* = 0.044), respectively. For patients with ER negative status, N39-positive patients had a marginally increased risk for recurrence (1.59-FI, *P* = 0.057), while for the ER positive group, N39-positive patients exhibited significantly increased risk for recurrence, 2.7-FI (*P* = 0.004). Breast cancer is strongly related to age with ~80% of breast cancer occurring in women age >50. We demonstrated that N39-positive women age <50 exhibit a 1.9-FI (*P* = 0.020) whereas women age >50 exhibit a 2.64-FI increased risk for recurrence (*P* = 8.4 × 10^−4^). Kaplan-Meier survival curves confirmed a significantly reduced survival for N39-positive patients in each subset grouped by age, lymph node status, tumor size, and ER status (Figure 5B).

## Discussion

*NAMPT* is a novel cancer marker^6^ and therapeutic target^26^ with unclear mechanisms of action. Regardless of intrinsic complex biologically function with differential roles as secreted proinflammatory cytokine (extracellular *NAMPT*) or rate-limiting NAD^+^ synthesis enzyme (intracellular *NAMPT*), we looked into the prognostic power with the gene sets regulated by *NAMPT*. Firstly we confirmed the critical role of *NAMPT* in carcinogenesis by the gene ontology analysis of all *NAMPT*-mediated genes: eight of the eleven significantly deregulated pathways are direct cancer pathways (Fig. 1). Secondly, we generated the N39 signature by filtering through gene express data sets of four cancer types. Thirdly, we validated N39 signature as a powerful tool to provide important prognostic lung and breast cancer and determined the N39 gene signature as a significant and independent predictor of cancer recurrence-free survival.

We chose lung and breast cancers to serve as the validation study for cancer survival prognosis, mainly dependent on the availability of the datasets (three independent studies to serve as one discovery cohort and two validation cohorts). Moreover, this choice of cancer type selection is based on the severity of the two types of cancer. Lung cancer is the most frequently diagnosed cancers and leading cause of cancer death in males, comprising 17% of the total new cancer cases and 23% of the total cancer deaths^27^. In females, breast cancer is the most frequently diagnosed cancer and the leading cause of cancer death, accounting for 23% of the total cancer cases and 14% of the cancer deaths^27^.

Prognostic molecular signatures that work cooperatively with traditional clinical and pathological factors may increase prognostic accuracy when identifying patients at higher risk for recurrence and death^28^,^29^,^30^. Our proposed molecular signature that is composed of 39 *NAMPT*-mediated genes is a promising prognostic marker, because N39 was solely developed based on the discovery cohort and its prognostic power was validated in two independent validation cohorts for lung and breast cancer, respectively. More importantly, N39 was independent of other clinicopathological covariates. In the Lung1 cohort, when grouped by age, *EGFR*/*KRAS*/*ALK* alteration status, and smoking history, N39 further stratified lung cancer patients with significant differences in survival. A significantly increased risk of recurrence was also observed in N39-positive patients of stage I. However, we failed to observe a significant difference between N39-positive and -negative groups among the patients of stage II, which may be due to the relatively smaller sample size in this category. To validate the prognostic power of N39 in stage II tumor, we further included an additional lung cancer dataset (GSE41271)^31^ here. We merged the subjects of stage II from three independent cohorts (Lung1, Lung2, and GSE41271) using the “metaArray” package in Bioconductor (see Supplementary Fig. S5 for details). We found that N39-positive patients (with stage II tumor) exhibited a significantly increased risk (1.68-FI, *P* = 0.049 by univariate Cox proportional hazards regression) for recurrence comparing with N39-negative patients. Also, Kaplan-Meier survival curves confirmed a significantly reduced survival (*P* = 0.047 by log-rank test) for N39-positive patients of stage II (Supplementary Fig. S5). In the Breast1 cohort, we stratified the patients according to age, lymph node status, tumor size, and ER status, respectively. A significantly increased risk of recurrence was also observed in N39-positive patients in each category, except for the marginal signal in ER negative patients. Taken together, these results confirm that N39 is not dependent on specific values of the respective covariates status, which enhances the identification of cancer patients at greater risk for recurrence.

We used the median of N39 risk score as a cutoff to stratify patents into two groups (N39-positive and -negative) to conduct categorized statistical analyses (such as Kaplan-Meier analysis and log-rank test). Clinically, zero can be utilized as an absolute cutoff to divide patients into high- and low-risk groups, as the median of N39 score is approximately equal to zero in each validation cohort (Supplementary Fig. S6).

In addition to its prognosis utility, N39 gene list also provides a set of *NAMPT* associated genes that might play critical roles in cancer pathogenesis. One good example is *SIRT1*. *NAMPT*-*SIRT1*-*MYC* axis critically regulates cell survival^32^. *SIRT1* is also found over-expressed in many cancers and frequently *NAMPT* is concurrently over-expressed with *SIRT1*, which is important for prostate cancer cell survival and stress response^33^. A recent study in pancreatic cancer lines, however, suggested that NADase CD38 but not *SIRT1* is crucial for pancreatic cancer cells' response to *NAMPT* inhibition^34^, suggesting the complex interaction of *NAMPT* with *SIRT1*. These previous findings, together with N39 signature, have generated novel biomarkers or therapeutic targets in cancer.

This study is an example of re-examination of available genomic/genetic data in the “big data” era, with a novel translational approach. *NAMPT* is confirmed to be a novel “oncogene” with a central role in carcinogenesis despite a clear molecular mechanism elucidated. In addition to cancer prognosis, now well validated in the current study, N39 has promise in the management of multiple cancers.

## Methods

### Expression microarray data

We obtained the gene expression data of WT and *NAMPT* KD MCF-7 breast cancer cells (GSE13449)^13^ and of WT and *NAMPT* KD pulmonary microvascular endothelial cells (GSE34512)^14^ from the NCBI GEO database^12^. The gene expression data of paired normal and tumor tissues for lung (GSE18842)^16^, colon (GSE23878)^17^, pancreatic (GSE15471)^18^, and thyroid (GSE33630) cancers were also collected from the GEO database. Training and validation cohorts were constructed for lung and breast cancers. From the GEO database, we collected the expression datasets with available information on recurrence-free survival for lung (GSE8894^19^ for training and GSE31210^21^ and GSE37745^22^ for validation) and breast (GSE2034^20^ for training and GSE25066^23^ and GSE21653^24^ for validation) cancers.

### Microarray data processing

The GC robust multichip average algorithm^35^ was used to summarize the expression level of each probe set for the microarray data of WT and *NAMPT* KD human cells and of paired normal and tumor tissues. Only the probe sets present (determined by function “mas5calls” in the Bioconductor “affy” package^36^) in at least two thirds of the samples were retained. We further limited our analysis to the probe sets with unique annotations and removed genes on chromosomes X and Y to avoid potential confounding factors. Significance analysis of microarrays^37^, implemented in the samr library of the R Statistical Package, was used to compare log_2_-transformed gene expression levels between WT and *NAMPT* KD human cells. FDR was controlled using the q-value method^38^,^39^. Transcripts with a fold-change greater than 1.1 and FDR less than 0.05 were deemed differentially expressed.

### Risk scoring system

For each training cohort, univariate Cox proportional hazards regression was used to evaluate the association between recurrence-free survival and gene expression. A risk score was then calculated for each patient using a linear combination of gene expression weighted by the Wald statistic (ratio of regression coefficient to its standard error) as shown below: 
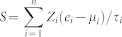


In the equation above, *S* is the risk score of patient; *n* is the number of genes; *Z_i_* denotes the Wald statistic of gene *i*; *e_i_* denotes the expression level of gene *i*; and *μ_i_* and *τ_i_* are the mean and standard deviation of the gene expression values for gene *i* across all samples, respectively. Patients were then divided into positive and negative groups with the median of the risk score as the threshold. A higher risk score implies a poor outcome. The scoring system and the associated scaling coefficients were fixed based on the training cohorts and then evaluated in the validation cohorts. All statistical analyses were conducted using the R platform (version 2.15.1). The *α* level for all the statistical tests was 0.05.

## Author Contributions

T.Z., T.W. and J.G.N.G. conceived of the study. T.Z., T.W. and J.G.N.G. participated in the design of the study. T.Z. and T.W. collected the microarray data. T.Z. and T.W. processed the microarray data. T.Z. and T.W. performed the statistical analysis. J.G.N.G. helped to interpret the results. T.Z., T.W. and J.G.N.G. drafted the manuscript. All authors read and approved the final manuscript.

## Supplementary Material

Supplementary Information Supplementary figures and tables

Supplementary Information Supplementary table S1

## Figures and Tables

**Table 1:** N39 gene set

Gene symbol	Gene title
*ADK*	adenosine kinase
*AP2B1*	adaptor-related protein complex 2, beta 1 subunit
*AVL9*	AVL9 homolog (S. cerevisiase)
*CANX*	calnexin
*DBT*	dihydrolipoamide branched chain transacylase E2
*DHRS7*	dehydrogenase/reductase (SDR family) member 7
*DONSON*	downstream neighbor of SON
*FAM190B*	family with sequence similarity 190, member B
*FGFR1*	fibroblast growth factor receptor 1
*FOXN3*	forkhead box N3
*FZD5*	frizzled family receptor 5
*GGH*	gamma-glutamyl hydrolase (conjugase, folylpolygammaglutamyl hydrolase)
*GM2A*	GM2 ganglioside activator
*IGFBP5*	insulin-like growth factor binding protein 5
*ITSN2*	intersectin 2
*LAMC1*	laminin, gamma 1 (formerly LAMB2)
*LIFR*	leukemia inhibitory factor receptor alpha
*METTL7A*	methyltransferase like 7A
*MT1F*	metallothionein 1F
*MT1G*	metallothionein 1G
*MT1P2*	metallothionein 1 pseudogene 2
*MT1X*	metallothionein 1X
*MT2A*	metallothionein 2A
*NAB1*	NGFI-A binding protein 1 (EGR1 binding protein 1)
*NCOA1*	nuclear receptor coactivator 1
*NCOR1*	nuclear receptor corepressor 1
*PAPOLA*	poly(A) polymerase alpha
*PPME1*	protein phosphatase methylesterase 1
*PPP1R13L*	protein phosphatase 1, regulatory subunit 13 like
*PRKAR2A*	protein kinase, cAMP-dependent, regulatory, type II, alpha
*RABEP1*	rabaptin, RAB GTPase binding effector protein 1
*RBBP8*	retinoblastoma binding protein 8
*SGPL1*	sphingosine-1-phosphate lyase 1
*SIRT1*	sirtuin 1
*SNX2*	sorting nexin 2
*SREK1*	splicing regulatory glutamine/lysine-rich protein 1
*TAF1B*	TATA box binding protein (TBP)-associated factor, RNA polymerase I, B, 63 kDa
*TMED5*	transmembrane emp24 protein transport domain containing 5
*ZMIZ2*	zinc finger, MIZ-type containing 2

**Table 2:** Cox proportional hazards regression of survival by N39 status in lung and breast cancers

	Training cohort				Validation cohort				
Cancer	n	HR	95% CI	*P*-value	Cohort	n	HR	95% CI	*P*-value
Lung	138	3.18	(1.91, 5.29)	8.4 × 10^−6^	Lung1	226	2.88	(1.69, 4.95)	1.2 × 10^−4^
					Lung2	96	2.08	(1.17, 3.70)	1.3 × 10^−2^
Breast	286	2.76	(1.83, 4.14)	1.1 × 10^−6^	Breast1	508	2.27	(1.53, 3.37)	4.8 × 10^−5^
					Breast2	252	2.12	(1.36, 3.30)	9.5 × 10^−4^

Note – n: sample size; HR: hazard ratio; CI: confidence interval.

**Table 3:** Multivariate Cox proportional hazards regression of survival in the validation cohorts

Cohort	Covariate	HR	95% CI	*P*-value
Lung1	N39 + vs. −	2.51	(1.44, 4.37)	1.2 × 10^−3^
	Age (per year)	1.04	(1.00, 1.08)	2.9 × 10^−2^
	Gender male vs. female	0.70	(0.35, 1.41)	3.2 × 10^−1^
	Smoking + vs. −	1.40	(0.72, 2.75)	3.2 × 10^−1^
	Stage	2.86	(1.68, 4.85)	9.8 × 10^−5^
	*EGFR*/*KRAS*/*ALK* alteration + vs. −	0.57	(0.34, 0.96)	3.6 × 10^−2^
	MYC level high vs. low	0.90	(0.35, 2.32)	8.3 × 10^−1^
Lung2	N39 + vs. −	2.32	(1.27, 4.25)	6.4 × 10^−3^
	Age (per year)	1.00	(0.97, 1.04)	8.0 × 10^−1^
	Gender male vs. female	0.85	(0.47, 1.54)	5.9 × 10^−1^
	Stage	1.44	(0.99, 2.09)	5.5 × 10^−2^
Breast1	N39 + vs. −	1.97	(1.26, 3.07)	2.9 × 10^−3^
	Age (per year)	1.00	(0.98, 1.02)	9.6 × 10^−1^
	Lymph node + vs. −	2.88	(1.66, 5.00)	1.8 × 10^−4^
	Grade 3 vs. 1,2	0.73	(0.45, 1.18)	2.0 × 10^−1^
	Tumor size ≥T3 vs. < T3	1.65	(1.11, 2.46)	1.3 × 10^−2^
	ER + vs. −	0.52	(0.30, 0.90)	2.0 × 10^−2^
	PR + vs. −	0.66	(0.39, 1.14)	1.4 × 10^−1^
Breast2	N39 + vs. −	1.97	(1.13, 3.43)	1.7 × 10^−2^
	Age (per year)	1.00	(0.98, 1.02)	8.5 × 10^−1^
	Grade 3 vs. 1,2	0.92	(0.52, 1.63)	7.7 × 10^−1^
	ER + vs. −	0.70	(0.26, 1.86)	4.7 × 10^−1^
	PR + vs. −	1.35	(0.52, 3.50)	5.4 × 10^−1^
	*TP53* alteration + vs. −	1.48	(0.87, 2.51)	1.4 × 10^−1^

Note – HR: hazard ratio; CI: confidence interval.
