# Supplementary material for: Expression of Nicotinamide Phosphoribosyltransferase-Influenced Genes Predicts Recurrence-Free Survival in Lung and Breast Cancers
Source: Sci Rep. 2014 Aug 22;4:6107. doi: 10.1038/srep06107 (PMC4141256; doi:10.1038/srep06107)
Supplement: Supplementary Information — Supplementary figures and tables [file srep06107-s1.pdf]

Supplementary materials

Expression of Nicotinamide Phosphoribosyltransferase-Influenced Genes Predicts Recurrence-Free Survival in Lung and Breast Cancers

Tong Zhou†, Ting Wang†, Joe G. N. Garcia*

Arizona Respiratory Center and Department of Medicine, The University of Arizona, Tucson, Arizona, USA

† These authors contributed equally to this work.

* Corresponding author: Joe G.N. Garcia, MD, Senior Vice President for Health Sciences, Arizona Health Sciences Center, The University of Arizona, Tel: (520) 626-1197, email: skipgarcia@email.arizona.edu

Funding: This work was supported by National Institutes of Health Grants HL094394 (JGNG).

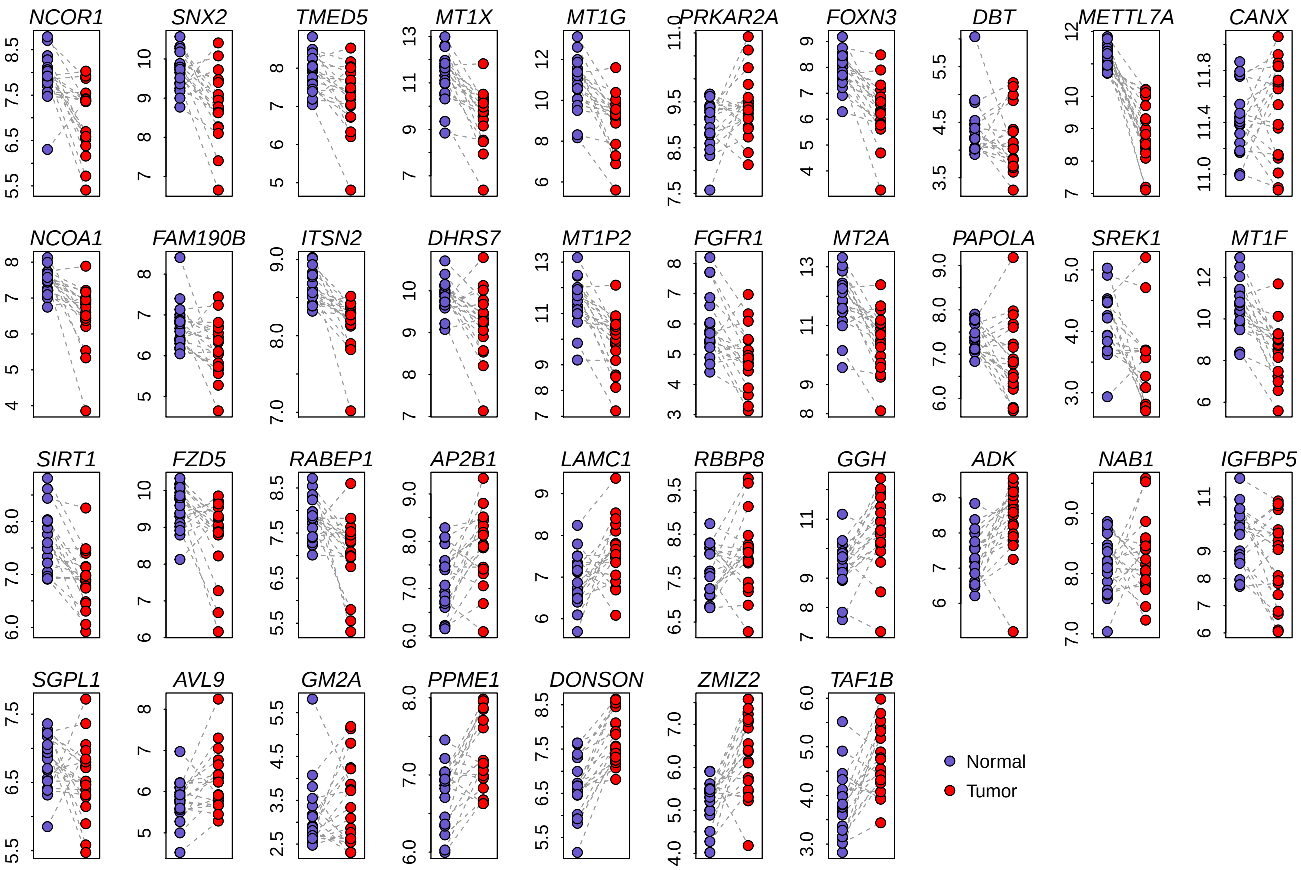

Figure S1. Comparison of N39 gene expression between normal and tumor tissues in colon cancer. Paired normal and tumor tissues from 19 colon cancer patients were included in the comparison. Two genes (*LIFR* and *PPP1R13L*) in N39 are not included in this figure because of absent call. All the listed genes are differentially expressed between normal and tumor tissues except *CANX*, *DBT*, *GM2A*, *IGFBP5*, *NAB1*, *PAPOLA*, *PRKAR2A*, *RBBP8*, and *SGPL1*. Y-axis: log_2-transformed expression values.

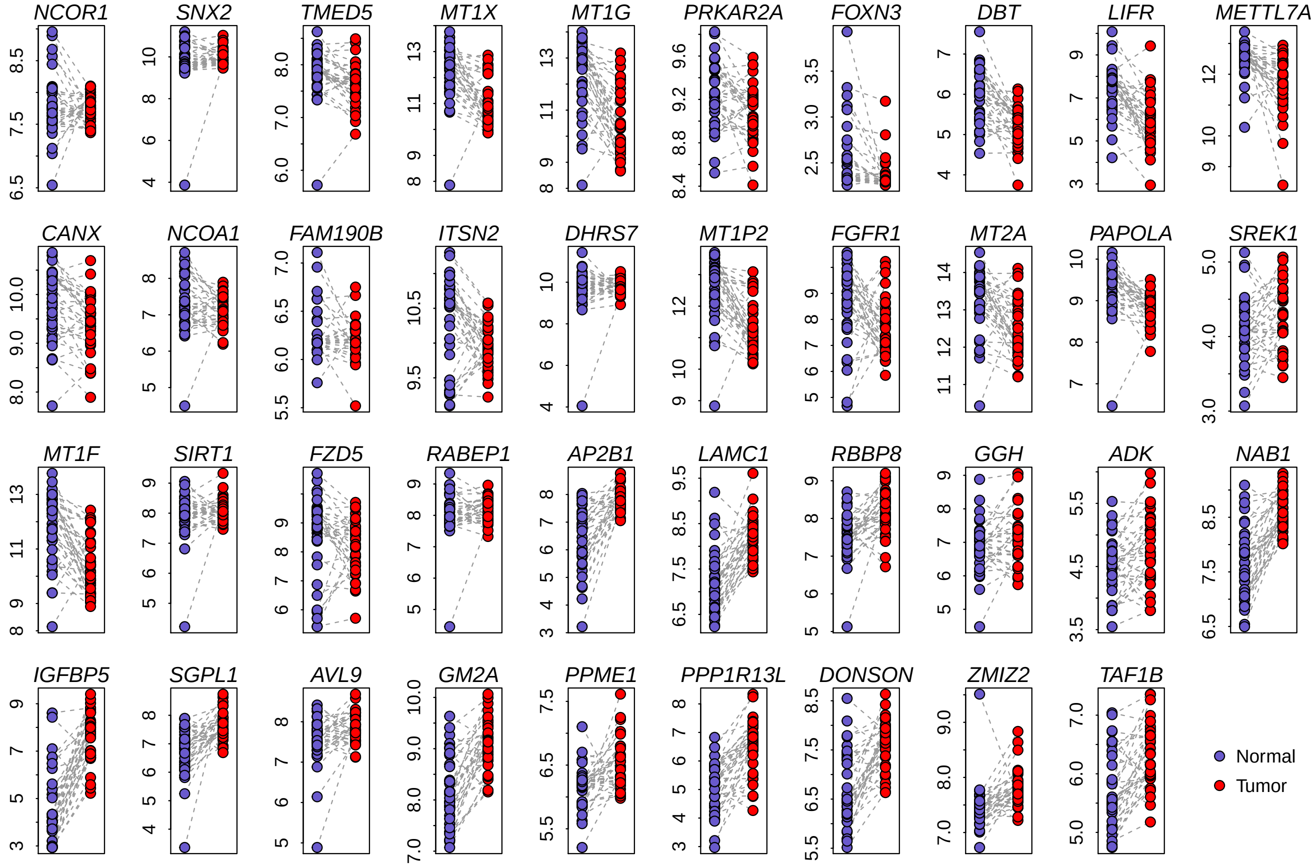

Figure S2. Comparison of N39 gene expression between normal and tumor tissues in pancreatic cancer. Paired normal and tumor tissues from 36 pancreatic cancer patients were included in the comparison. All the listed genes are differentially expressed between normal and tumor tissues except *DHRS7*, *FAM190B*, *FZD5*, *NCOA1*, *NCOR1*, *RABEP1*, *SIRT1*, *SNX2*, and *SREK1*. Y-axis: log_2-transformed expression values.

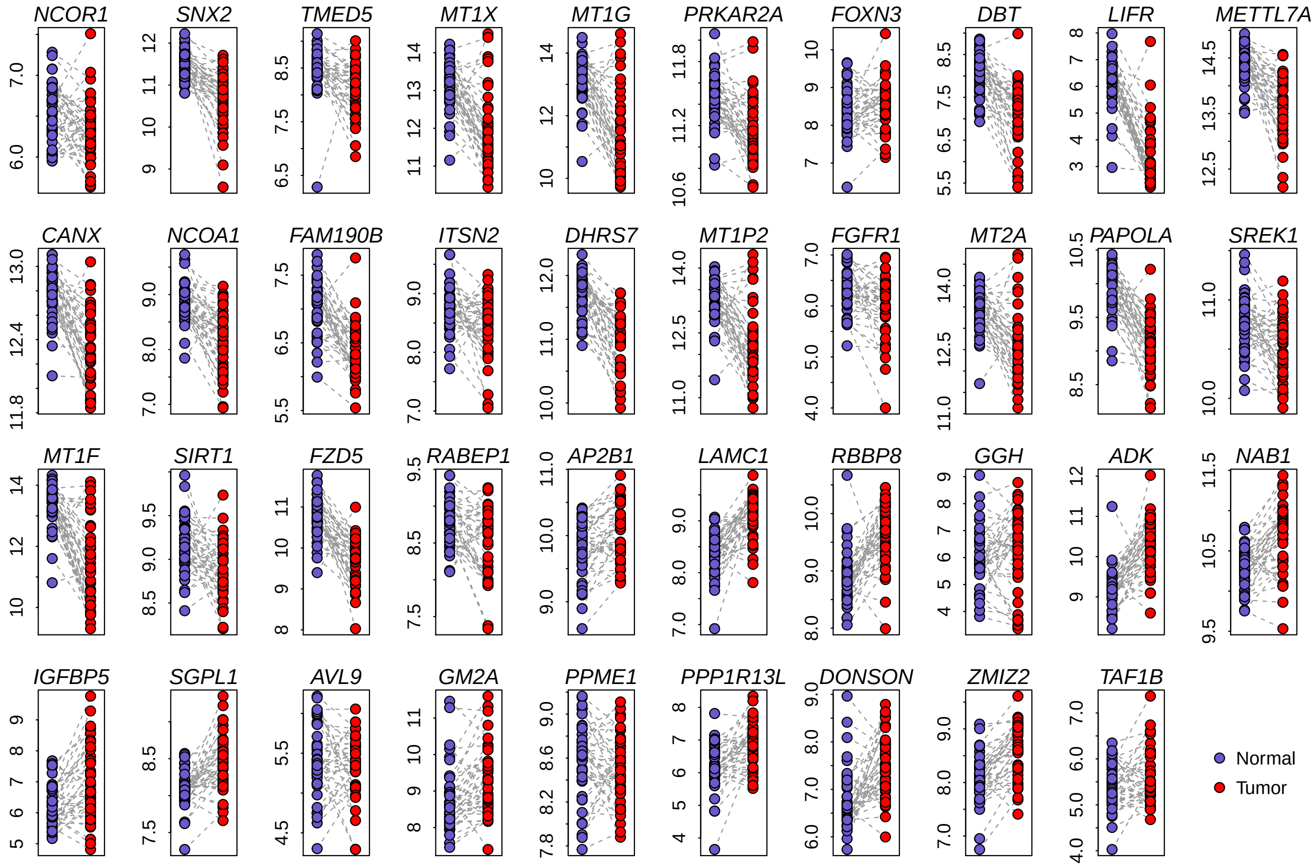

Figure S3. Comparison of N39 gene expression between normal and tumor tissues in thyroid cancer. Paired normal and tumor tissues from 44 thyroid cancer patients were included in the comparison. All the listed genes are differentially expressed between normal and tumor tissues except *AVL9*, *FOXN3*, *GGH*, *PPME1*, and *TAF1B*. Y-axis: log_2-transformed expression values.

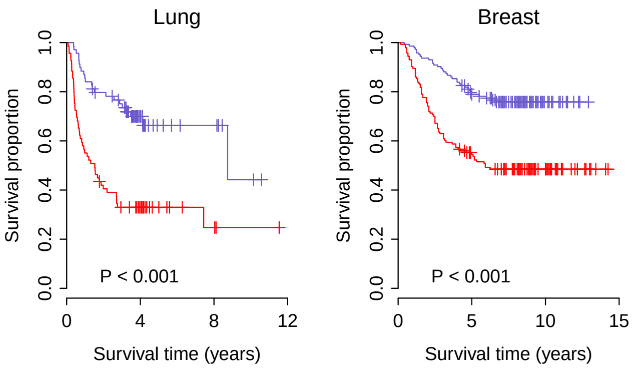

Figure S4. Kaplan-Meier curves for patients in training cohorts. The expression of N39 predicts poor recurrence-free survival in training cohorts of lung and breast cancers. Red curves are for N39-positive patients while blue curves are for N39-negative patients. N39-positive patients were defined as those having a risk score greater than the group median. P-values were calculated by log-rank tests for the differences in survival between N39-positive and -negative groups.

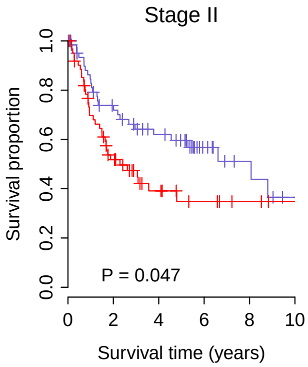

Figure S5. Kaplan-Meier curves for lung cancer patients with stage II tumor. The expression of N39 predicts poor recurrence-free survival in lung cancer patients with stage II tumor from three different cohorts (Lung1, Lung2, and GSE41271). We merged the subjects with stage II tumor (126 in total) from these three studies for meta-analysis to ensure sufficient statistical power. We normalized the gene expression level into the scale of [-1, 1] by Probability of Expression (POE) algorithm implemented in the metaArray package, which transforms gene expression data to signed probability scale for meta-analysis of microarray data. Red curves are for N39-positive patients while blue curves are for N39-negative patients. N39-positive patients were defined as those having a risk score greater than the group median. P-values were calculated by log-rank tests for the differences in survival between N39-positive and -negative groups.

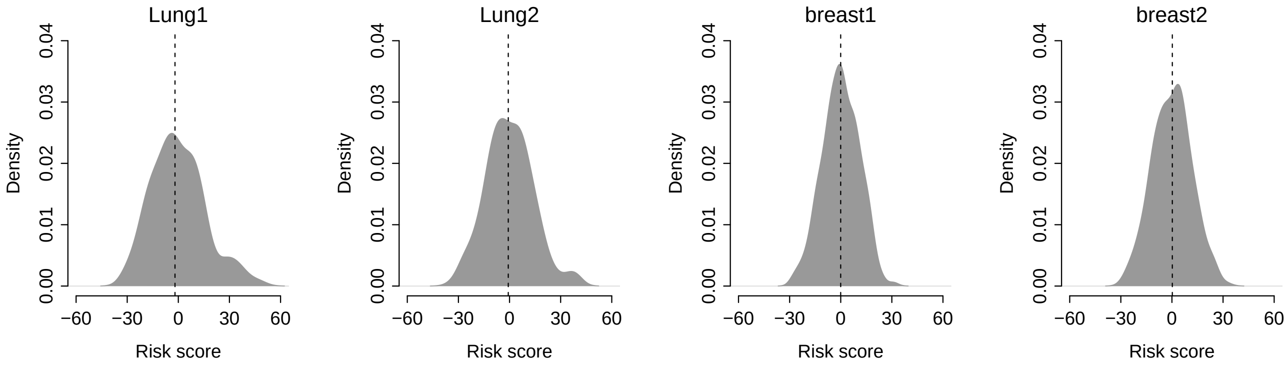

Figure S6. Distribution of N39 risk score in validation cohorts. The vertical dash line indicates the median of risk score.

Table S1. Common differentially expressed genes between WT and *NAMPT* knock down cells

		GSE13449		GSE34512	
Gene symbol	Gene title	Fold change	FDR (%)	Fold change	FDR (%)
AASDHPPT	aminoadipate-semialdehyde dehydrogenase-phosphopantetheinyl transferase	1.31	4.09	2.52	0.00
ABAT	4-aminobutyrate aminotransferase	1.28	4.09	1.45	1.04
ABCD3	ATP-binding cassette, sub-family D (ALD), member 3	0.91	4.09	0.83	2.90
ABCE1	ATP-binding cassette, sub-family E (OABP), member 1	1.16	4.09	1.49	0.00
ABHD10	abhydrolase domain containing 10	0.89	4.09	0.75	2.90
ABI2	abl-interactor 2	0.84	0.00	0.81	3.72
ACO1	aconitase 1, soluble	0.90	4.09	0.59	3.72
ACSL3	acyl-CoA synthetase long-chain family member 3	1.43	4.09	1.27	0.00
ADAM17	ADAM metallopeptidase domain 17	1.15	4.09	1.16	0.00
ADK	adenosine kinase	0.81	0.00	0.78	3.72
AGTPBP1	ATP/GTP binding protein 1	0.81	4.09	0.69	2.90
ALG9	asparagine-linked glycosylation 9, alpha-1,2-mannosyltransferase homolog (S. cerevisiae)	0.82	4.09	0.80	2.90
ANAPC13	anaphase promoting complex subunit 13	0.90	0.00	0.84	2.90
ANKRD10	ankyrin repeat domain 10	1.26	4.09	1.55	0.00
ANKRD17	ankyrin repeat domain 17	1.19	4.09	1.50	0.00
ANKRD27	ankyrin repeat domain 27 (VPS9 domain)	1.13	4.09	1.27	0.00
ANP32E	acidic (leucine-rich) nuclear phosphoprotein 32 family, member E	1.48	4.09	1.14	1.04
AP2B1	adaptor-related protein complex 2, beta 1 subunit	0.89	0.00	0.54	2.90
AP4E1	adaptor-related protein complex 4, epsilon 1 subunit	1.20	4.09	1.50	0.00
ARHGEF2	Rho/Rac guanine nucleotide exchange factor (GEF) 2	1.55	4.09	1.89	0.00
ARHGEF3	Rho guanine nucleotide exchange factor (GEF) 3	1.33	4.09	1.39	0.00
ARL6IP1	ADP-ribosylation factor-like 6 interacting protein 1	1.18	4.09	1.74	0.00
ASB8	ankyrin repeat and SOCS box containing 8	0.90	0.00	0.52	2.90
ASF1A	ASF1 anti-silencing function 1 homolog A (S. cerevisiae)	1.21	4.09	1.44	0.00
ASUN	asunder, spermatogenesis regulator homolog (Drosphila)	1.21	4.09	1.78	0.00
ATF7IP	activating transcription factor 7 interacting protein	1.45	4.09	2.03	0.00
ATP10D	ATPase, class V, type 10D	0.77	4.09	0.76	2.90
ATP2C1	ATPase, Ca++ transporting, type 2C, member 1	1.24	4.09	1.49	0.00
ATXN1	ataxin 1	0.81	0.00	0.79	2.90
ATXN10	ataxin 10	2.24	4.09	1.38	0.00
AVL9	AVL9 homolog (S. cerevisiase)	0.90	0.00	0.52	2.90
AVPI1	arginine vasopressin-induced 1	1.24	4.09	1.34	1.04
BAX	BCL2-associated X protein	1.19	4.09	2.12	0.00
BAZ1A	bromodomain adjacent to zinc finger domain, 1A	1.38	4.09	1.24	0.00
BAZ2A	bromodomain adjacent to zinc finger domain, 2A	1.12	4.09	2.02	0.00
BBX	bobby sox homolog (Drosophila)	1.45	4.09	1.34	0.00
BCAR3	breast cancer anti-estrogen resistance 3	1.21	4.09	1.39	0.00
BCAT1	branched chain amino-acid transaminase 1, cytosolic	1.21	4.09	1.30	1.04
BLVRB	biliverdin reductase B (flavin reductase (NADPH))	1.20	4.09	1.74	0.00
BMPR2	bone morphogenetic protein receptor, type II (serine/threonine kinase)	1.72	4.09	1.57	0.00
BST2	bone marrow stromal cell antigen 2	1.27	4.09	1.46	0.00
BTBD7	BTB (POZ) domain containing 7	0.81	0.00	0.88	2.90

Gene symbol	Gene title	GSE13449 Fold change	GSE13449 FDR (%)	GSE34512 Fold change	GSE34512 FDR (%)
BTG3	BTG family, member 3	0.67	0.00	0.75	2.90
C10orf57	chromosome 10 open reading frame 57	0.78	0.00	0.91	3.72
C12orf29	chromosome 12 open reading frame 29	0.89	0.00	0.82	2.90
C12orf35	chromosome 12 open reading frame 35	1.30	4.09	1.45	0.00
C12orf51	chromosome 12 open reading frame 51	1.12	4.09	1.64	0.00
C1orf115	chromosome 1 open reading frame 115	1.38	4.09	1.31	1.04
C1orf63	chromosome 1 open reading frame 63	1.16	4.09	1.69	0.00
C21orf91	chromosome 21 open reading frame 91	0.89	4.09	0.89	3.61
CAND1	cullin-associated and neddylation-dissociated 1	1.11	4.09	2.77	0.00
CANX	calnexin	1.19	4.09	1.24	0.00
CAP2	CAP, adenylate cyclase-associated protein, 2 (yeast)	1.27	4.09	1.14	0.00
CAPN1	calpain 1, (mu/I) large subunit	1.13	4.09	1.49	1.04
CASP4	caspase 4, apoptosis-related cysteine peptidase	1.18	4.09	1.26	1.04
CAST	calpastatin	1.97	4.09	1.21	0.00
CCDC47	coiled-coil domain containing 47	1.40	4.09	1.14	0.00
CCDC85C	coiled-coil domain containing 85C	1.11	4.09	1.27	1.04
CCNB1	cyclin B1	1.21	4.09	1.68	0.00
CCND1	cyclin D1	1.19	4.09	1.45	0.00
CD47	CD47 molecule	1.12	4.09	1.69	0.00
CDK13	cyclin-dependent kinase 13	1.23	4.09	1.45	0.00
CDK14	cyclin-dependent kinase 14	0.44	0.00	0.81	3.72
CDKN2C	cyclin-dependent kinase inhibitor 2C (p18, inhibits CDK4)	0.83	0.00	0.73	2.90
CDV3	CDV3 homolog (mouse)	1.16	4.09	1.19	0.00
CEP104	centrosomal protein 104kDa	1.39	4.09	1.41	0.00
CEP350	centrosomal protein 350kDa	1.25	4.09	1.52	0.00
CHKA	choline kinase alpha	1.25	4.09	1.72	0.00
CHMP1B	charged multivesicular body protein 1B	0.83	0.00	0.85	3.72
CKAP4	cytoskeleton-associated protein 4	1.46	4.09	1.40	0.00
CNOT4	CCR4-NOT transcription complex, subunit 4	1.10	4.09	1.44	0.00
COG7	component of oligomeric golgi complex 7	1.13	4.09	1.40	1.04
COQ10B	coenzyme Q10 homolog B (S. cerevisiae)	1.27	4.09	1.23	0.00
CPNE1	copine I	1.23	4.09	2.43	0.00
CPNE3	copine III	1.11	4.09	1.29	0.00
CREBL2	cAMP responsive element binding protein-like 2	0.81	0.00	0.90	3.72
CROT	carnitine O-octanoyltransferase	1.14	4.09	1.33	0.00
CSRP2	cysteine and glycine-rich protein 2	0.61	0.00	0.80	3.72
CTBP2	C-terminal binding protein 2	1.16	4.09	1.14	0.00
CTNNB1	catenin (cadherin-associated protein), beta 1, 88kDa	1.11	4.09	1.25	0.00
CTSC	cathepsin C	0.46	0.00	0.81	2.90
CUL2	cullin 2	1.30	4.09	1.26	0.00
CUL3	cullin 3	1.10	4.09	1.27	0.00
CXADR	coxsackie virus and adenovirus receptor	0.77	4.09	0.62	2.90
CXCL12	chemokine (C-X-C motif) ligand 12	0.54	0.00	0.63	3.61
CYBRD1	cytochrome b reductase 1	0.80	4.09	0.70	2.90
DBT	dihydrolipoamide branched chain transacylase E2	1.13	4.09	1.24	1.04
DCLRE1C	DNA cross-link repair 1C	1.19	4.09	1.35	0.00
DDIT4	DNA-damage-inducible transcript 4	1.54	4.09	1.62	0.00

Gene symbol	Gene title	GSE13449 Fold change	GSE13449 FDR (%)	GSE34512 Fold change	GSE34512 FDR (%)
DDX18	DEAD (Asp-Glu-Ala-Asp) box polypeptide 18	1.24	4.09	1.10	0.00
DDX54	DEAD (Asp-Glu-Ala-Asp) box polypeptide 54	1.18	4.09	1.28	1.04
DHRS7	dehydrogenase/reductase (SDR family) member 7	1.30	4.09	1.43	0.00
DHX29	DEAH (Asp-Glu-Ala-His) box polypeptide 29	1.27	4.09	1.17	1.04
DHX8	DEAH (Asp-Glu-Ala-His) box polypeptide 8	0.84	0.00	0.90	3.72
DIP2C	DIP2 disco-interacting protein 2 homolog C (Drosophila)	1.28	4.09	1.72	0.00
DLGAP4	discs, large (Drosophila) homolog-associated protein 4	1.18	4.09	1.21	1.04
DNAJA2	DnaJ (Hsp40) homolog, subfamily A, member 2	1.15	4.09	1.40	0.00
DNAJC2	DnaJ (Hsp40) homolog, subfamily C, member 2	1.35	4.09	1.32	0.00
DNTTIP2	deoxynucleotidyltransferase, terminal, interacting protein 2	1.22	4.09	1.18	1.04
DOCK9	dedicator of cytokinesis 9	1.14	4.09	1.12	0.00
DONSON	downstream neighbor of SON	0.73	0.00	0.80	2.90
DPP8	dipeptidyl-peptidase 8	1.18	4.09	1.35	0.00
DRAM1	DNA-damage regulated autophagy modulator 1	1.34	4.09	1.14	1.04
DYRK2	dual-specificity tyrosine-(Y)-phosphorylation regulated kinase 2	0.81	0.00	0.66	2.90
EFEMP1	EGF containing fibulin-like extracellular matrix protein 1	1.23	4.09	1.18	3.24
EFNA1	ephrin-A1	0.91	0.00	0.82	2.90
EIF2S1	eukaryotic translation initiation factor 2, subunit 1 alpha, 35kDa	1.11	4.09	1.28	0.00
EIF4E	eukaryotic translation initiation factor 4E	1.15	4.09	2.09	0.00
EIF4EBP2	eukaryotic translation initiation factor 4E binding protein 2	0.85	0.00	0.87	3.72
EIF4G1	eukaryotic translation initiation factor 4 gamma, 1	1.13	4.09	1.56	1.04
EIF5B	eukaryotic translation initiation factor 5B	1.74	4.09	1.38	0.00
ELOVL6	ELOVL fatty acid elongase 6	1.23	4.09	1.13	1.04
ENO1	enolase 1, (alpha)	1.13	4.09	1.34	0.00
ENO2	enolase 2 (gamma, neuronal)	0.91	0.00	0.51	3.72
ENSA	endosulfine alpha	1.12	4.09	1.35	0.00
EP300	E1A binding protein p300	1.21	4.09	1.14	0.00
EPHB4	EPH receptor B4	1.38	4.09	1.91	0.00
EPS15	epidermal growth factor receptor pathway substrate 15	1.58	4.09	16.59	0.00
EPS8	epidermal growth factor receptor pathway substrate 8	0.86	0.00	0.67	2.90
EXT1	exostosin 1	0.69	0.00	0.56	2.90
EZH2	enhancer of zeste homolog 2 (Drosophila)	0.83	4.09	0.41	2.90
EZR	ezrin	1.18	4.09	2.42	0.00
FABP5	fatty acid binding protein 5 (psoriasis-associated)	1.21	4.09	1.19	0.00
FAM134A	family with sequence similarity 134, member A	0.74	0.00	0.88	3.72
FAM172A	family with sequence similarity 172, member A	0.76	0.00	0.68	2.90
FAM178A	family with sequence similarity 178, member A	1.70	4.09	1.60	0.00
FAM190B	family with sequence similarity 190, member B	1.40	4.09	1.49	1.04
FAM46A	family with sequence similarity 46, member A	1.33	4.09	1.91	0.00
FARP1	FERM, RhoGEF (ARHGEF) and pleckstrin domain protein 1 (chondrocyte-derived)	0.89	4.09	0.56	2.90
FBXO42	F-box protein 42	1.13	4.09	1.22	3.24
FGFR1	fibroblast growth factor receptor 1	1.15	4.09	1.21	3.24

Gene symbol	Gene title	GSE13449 Fold change	GSE13449 FDR (%)	GSE34512 Fold change	GSE34512 FDR (%)
FH	fumarate hydratase	0.90	0.00	0.74	2.90
FLII	flightless I homolog (Drosophila)	1.18	4.09	1.26	1.04
FLNB	filamin B, beta	0.86	0.00	0.76	2.90
FNBP1	formin binding protein 1	1.19	4.09	1.34	0.00
FOXN3	forkhead box N3	1.10	4.09	1.56	0.00
FRY	furry homolog (Drosophila)	1.23	4.09	1.77	0.00
FUBP1	far upstream element (FUSE) binding protein 1	1.25	4.09	1.67	0.00
FXN	frataxin	1.38	4.09	1.40	1.04
FXR1	fragile X mental retardation, autosomal homolog 1	1.17	4.09	1.22	0.00
FZD5	frizzled family receptor 5	1.15	4.09	6.75	0.00
FZD6	frizzled family receptor 6	1.13	4.09	1.55	0.00
GALC	galactosylceramidase	0.70	4.09	0.55	2.90
GALK2	galactokinase 2	1.52	4.09	1.40	0.00
GALNT1	UDP-N-acetyl-alpha-D-galactosamine:polypeptide N-acetylgalactosaminyltransferase 1 (GalNAc-T1)	1.12	4.09	1.21	0.00
GALNT12	UDP-N-acetyl-alpha-D-galactosamine:polypeptide N-acetylgalactosaminyltransferase 12 (GalNAc-T12)	1.13	4.09	1.36	0.00
GALNT6	UDP-N-acetyl-alpha-D-galactosamine:polypeptide N-acetylgalactosaminyltransferase 6 (GalNAc-T6)	1.38	4.09	1.19	3.24
GAR1	GAR1 ribonucleoprotein homolog (yeast)	1.21	4.09	1.28	0.00
GATAD1	GATA zinc finger domain containing 1	1.36	4.09	1.67	0.00
GGH	gamma-glutamyl hydrolase (conjugase, folylpolygammaglutamyl hydrolase)	0.75	0.00	0.53	2.90
GGPS1	geranylgeranyl diphosphate synthase 1	0.69	0.00	0.84	2.90
GHITM	growth hormone inducible transmembrane protein	1.13	4.09	1.19	0.00
GIGYF2	GRB10 interacting GYF protein 2	1.17	4.09	1.73	0.00
GLRB	glycine receptor, beta	0.87	0.00	0.72	2.90
GM2A	GM2 ganglioside activator	0.74	0.00	0.79	2.90
GMCL1	germ cell-less homolog 1 (Drosophila)	0.66	0.00	0.90	2.90
GNAI3	guanine nucleotide binding protein (G protein), alpha inhibiting activity polypeptide 3	1.18	4.09	1.32	0.00
GNAQ	guanine nucleotide binding protein (G protein), q polypeptide	1.22	4.09	3.57	0.00
GNAS	GNAS complex locus	0.86	0.00	0.73	3.72
GNB1	guanine nucleotide binding protein (G protein), beta polypeptide 1	1.15	4.09	1.36	1.04
GNG5	guanine nucleotide binding protein (G protein), gamma 5	1.16	4.09	1.95	0.00
GRSF1	G-rich RNA sequence binding factor 1	1.11	4.09	1.41	0.00
GSK3B	glycogen synthase kinase 3 beta	1.22	4.09	1.52	0.00
GSPT1	G1 to S phase transition 1	1.20	4.09	1.71	0.00
HES1	hairy and enhancer of split 1, (Drosophila)	1.16	4.09	1.26	0.00
HEXIM1	hexamethylene bis-acetamide inducible 1	1.27	4.09	1.92	0.00
HIF1A	hypoxia inducible factor 1, alpha subunit (basic helix-loop-helix transcription factor)	1.25	4.09	1.30	0.00
HMGCS1	3-hydroxy-3-methylglutaryl-CoA synthase 1 (soluble)	1.41	4.09	1.85	0.00
HMGN1	high mobility group nucleosome binding domain 1	1.11	4.09	1.31	0.00
HMGXB4	HMG box domain containing 4	1.12	4.09	1.40	0.00
HN1	hematological and neurological expressed 1	1.13	4.09	1.43	0.00

Gene symbol	Gene title	GSE13449 Fold change	GSE13449 FDR (%)	GSE34512 Fold change	GSE34512 FDR (%)
HN1L	hematological and neurological expressed 1-like	0.87	4.09	0.51	2.90
HNRNPA2B1	heterogeneous nuclear ribonucleoprotein A2/B1	1.16	4.09	1.12	1.04
HNRNPH1	heterogeneous nuclear ribonucleoprotein H1 (H)	1.22	4.09	1.42	0.00
HNRNPM	heterogeneous nuclear ribonucleoprotein M	1.11	4.09	1.30	0.00
HNRNPR	heterogeneous nuclear ribonucleoprotein R	1.20	4.09	1.50	0.00
HNRNPU	heterogeneous nuclear ribonucleoprotein U (scaffold attachment factor A)	1.23	4.09	1.56	0.00
HSPA4	heat shock 70kDa protein 4	1.11	4.09	1.55	0.00
HSPA9	heat shock 70kDa protein 9 (mortalin)	1.22	4.09	1.70	0.00
HSPB1	heat shock 27kDa protein 1	1.12	4.09	1.40	1.04
HSPB11	heat shock protein family B (small), member 11	1.14	4.09	1.61	0.00
HSPBAP1	HSPB (heat shock 27kDa) associated protein 1	0.81	4.09	0.81	2.90
IBTK	inhibitor of Bruton agammaglobulinemia tyrosine kinase	1.21	4.09	2.78	0.00
IFI30	interferon, gamma-inducible protein 30	1.75	4.09	4.42	0.00
IFIH1	interferon induced with helicase C domain 1	1.50	4.09	2.40	0.00
IGFBP5	insulin-like growth factor binding protein 5	0.63	0.00	0.90	2.90
INSIG1	insulin induced gene 1	1.40	4.09	1.41	0.00
INTS6	integrator complex subunit 6	1.22	4.09	1.79	0.00
IPO9	importin 9	0.86	4.09	0.42	2.90
ITGAE	integrin, alpha E (antigen CD103, human mucosal lymphocyte antigen 1; alpha polypeptide)	0.87	0.00	0.74	2.90
ITGB4	integrin, beta 4	1.50	4.09	1.12	0.00
ITPKC	inositol-trisphosphate 3-kinase C	1.11	4.09	2.05	0.00
ITPR1	inositol 1,4,5-trisphosphate receptor, type 1	0.56	0.00	0.89	2.90
ITSN1	intersectin 1 (SH3 domain protein)	1.45	4.09	1.33	0.00
ITSN2	intersectin 2	1.39	4.09	1.36	0.00
IVD	isovaleryl-CoA dehydrogenase	1.25	4.09	1.82	0.00
JUP	junction plakoglobin	1.11	4.09	1.51	0.00
KCTD3	potassium channel tetramerisation domain containing 3	1.12	4.09	1.22	1.04
KDM3B	lysine (K)-specific demethylase 3B	1.25	4.09	1.40	1.04
KEAP1	kelch-like ECH-associated protein 1	1.17	4.09	1.45	1.04
KIAA1324	KIAA1324	0.82	0.00	0.60	2.90
KITLG	KIT ligand	1.45	4.09	1.21	0.00
KRIT1	KRIT1, ankyrin repeat containing	1.33	4.09	1.42	0.00
LAMC1	laminin, gamma 1 (formerly LAMB2)	0.90	0.00	0.76	3.72
LARP1	La ribonucleoprotein domain family, member 1	1.17	4.09	1.15	0.00
LARP4	La ribonucleoprotein domain family, member 4	1.25	4.09	1.34	0.00
LDHA	lactate dehydrogenase A	1.11	4.09	1.19	0.00
LEPROTL1	leptin receptor overlapping transcript-like 1	0.87	0.00	0.50	2.90
LGALS3BP	lectin, galactoside-binding, soluble, 3 binding protein	1.74	4.09	1.86	0.00
LIFR	leukemia inhibitory factor receptor alpha	1.33	4.09	3.25	0.00
LMNA	lamin A/C	1.19	4.09	1.56	1.04
LPCAT1	lysophosphatidylcholine acyltransferase 1	1.13	4.09	1.54	0.00
LPIN2	lipin 2	1.16	4.09	2.24	0.00
LRFN4	leucine rich repeat and fibronectin type III domain containing 4	1.11	4.09	1.82	0.00
LRP8	low density lipoprotein receptor-related protein 8, apolipoprotein e receptor	1.39	4.09	1.11	1.04

Gene symbol	Gene title	GSE13449 Fold change	GSE13449 FDR (%)	GSE34512 Fold change	GSE34512 FDR (%)
LRRFIP1	leucine rich repeat (in FLII) interacting protein 1	1.42	4.09	1.22	0.00
LSM14A	LSM14A, SCD6 homolog A (S. cerevisiae)	1.15	4.09	1.50	0.00
LUC7L3	LUC7-like 3 (S. cerevisiae)	1.26	4.09	1.27	0.00
LYRM1	LYR motif containing 1	1.31	4.09	1.26	0.00
MALT1	mucosa associated lymphoid tissue lymphoma translocation gene 1	1.19	4.09	2.19	0.00
MAP2K6	mitogen-activated protein kinase kinase 6	1.21	4.09	1.62	0.00
MARC2	mitochondrial amidoxime reducing component 2	0.87	0.00	0.89	2.90
MBNL2	muscleblind-like splicing regulator 2	1.54	4.09	1.37	0.00
MBOAT7	membrane bound O-acyltransferase domain containing 7	1.20	4.09	1.58	0.00
MCFD2	multiple coagulation factor deficiency 2	1.32	4.09	1.14	0.00
MED17	mediator complex subunit 17	0.90	0.00	0.77	3.61
MED6	mediator complex subunit 6	1.32	4.09	1.43	0.00
MEF2A	myocyte enhancer factor 2A	0.90	0.00	0.77	2.90
METTL7A	methyltransferase like 7A	1.25	4.09	2.18	0.00
MFHAS1	malignant fibrous histiocytoma amplified sequence 1	1.50	4.09	1.51	0.00
MGA	MAX gene associated	1.25	4.09	1.14	0.00
MGEA5	meningioma expressed antigen 5 (hyaluronidase)	1.24	4.09	1.21	0.00
MORC3	MORC family CW-type zinc finger 3	1.23	4.09	1.18	0.00
MRPS35	mitochondrial ribosomal protein S35	1.18	4.09	1.53	1.04
MRTO4	mRNA turnover 4 homolog (S. cerevisiae)	1.17	4.09	1.34	1.04
MSL1	male-specific lethal 1 homolog (Drosophila)	1.15	4.09	1.43	0.00
MT1F	metallothionein 1F	1.41	4.09	1.38	0.00
MT1G	metallothionein 1G	1.21	4.09	1.21	0.00
MT1P2	metallothionein 1 pseudogene 2	1.44	4.09	1.29	0.00
MT1X	metallothionein 1X	1.44	4.09	1.31	0.00
MT2A	metallothionein 2A	1.46	4.09	1.22	0.00
MTDH	metadherin	1.17	4.09	1.12	0.00
MTO1	mitochondrial translation optimization 1 homolog (S. cerevisiae)	1.18	4.09	1.18	0.00
MTR	5-methyltetrahydrofolate-homocysteine methyltransferase	1.43	4.09	2.21	0.00
MTUS1	microtubule associated tumor suppressor 1	1.34	4.09	3.09	0.00
MYOF	myoferlin	1.34	4.09	1.17	0.00
NAB1	NGFI-A binding protein 1 (EGR1 binding protein 1)	0.64	0.00	0.83	2.90
NACC2	NACC family member 2, BEN and BTB (POZ) domain containing	1.24	4.09	1.41	1.04
NAMPT	nicotinamide phosphoribosyltransferase	0.09	0.00	0.02	2.90
NBAS	neuroblastoma amplified sequence	0.88	4.09	0.85	3.72
NBN	nibrin	1.12	4.09	1.48	0.00
NCL	nucleolin	1.11	4.09	1.32	0.00
NCOA1	nuclear receptor coactivator 1	1.32	4.09	4.84	0.00
NCOR1	nuclear receptor corepressor 1	1.36	4.09	1.52	0.00
NET1	neuroepithelial cell transforming 1	1.14	4.09	2.37	0.00
NFATC2IP	nuclear factor of activated T-cells, cytoplasmic, calcineurin-dependent 2 interacting protein	1.15	4.09	3.54	0.00
NFIB	nuclear factor I/B	1.62	4.09	1.75	0.00
NFKBIA	nuclear factor of kappa light polypeptide gene enhancer in B-cells inhibitor, alpha	1.21	4.09	1.25	1.04

Gene symbol	Gene title	GSE13449 Fold change	GSE13449 FDR (%)	GSE34512 Fold change	GSE34512 FDR (%)
NINJ1	ninjurin 1	1.22	4.09	1.49	0.00
NLK	nemo-like kinase	1.38	4.09	1.26	0.00
NRCAM	neuronal cell adhesion molecule	0.67	0.00	0.56	2.90
NUPL1	nucleoporin like 1	1.65	4.09	1.58	0.00
NUSAP1	nucleolar and spindle associated protein 1	1.11	4.09	1.33	1.04
OPA1	optic atrophy 1 (autosomal dominant)	1.43	4.09	1.28	0.00
OPN3	opsin 3	1.14	4.09	1.66	0.00
OSBPL10	oxysterol binding protein-like 10	1.18	4.09	1.40	0.00
OSMR	oncostatin M receptor	1.74	4.09	2.90	0.00
PAICS	phosphoribosylaminoimidazole carboxylase, phosphoribosylaminoimidazole succinocarboxamide synthetase	1.18	4.09	4.75	0.00
PAK2	p21 protein (Cdc42/Rac)-activated kinase 2	1.15	4.09	1.88	0.00
PAPOLA	poly(A) polymerase alpha	1.19	4.09	1.57	0.00
PAPSS1	3'-phosphoadenosine 5'-phosphosulfate synthase 1	0.87	0.00	0.86	3.72
PAPSS2	3'-phosphoadenosine 5'-phosphosulfate synthase 2	0.57	0.00	0.78	3.72
PBX2	pre-B-cell leukemia homeobox 2	1.33	4.09	1.90	0.00
PCMT1	protein-L-isoaspartate (D-aspartate) O-methyltransferase	1.11	4.09	1.26	0.00
PDLIM7	PDZ and LIM domain 7 (enigma)	1.13	4.09	2.21	0.00
PHACTR2	phosphatase and actin regulator 2	1.51	4.09	1.24	0.00
PHF20L1	PHD finger protein 20-like 1	1.25	4.09	1.44	1.04
PHKB	phosphorylase kinase, beta	0.86	0.00	0.76	2.90
PHLDA3	pleckstrin homology-like domain, family A, member 3	1.13	4.09	1.60	0.00
PHTF1	putative homeodomain transcription factor 1	0.84	0.00	0.82	2.90
PIAS1	protein inhibitor of activated STAT, 1	1.20	4.09	1.66	0.00
PIK3R3	phosphoinositide-3-kinase, regulatory subunit 3 (gamma)	1.22	4.09	1.51	0.00
PIP4K2B	phosphatidylinositol-5-phosphate 4-kinase, type II, beta	1.60	4.09	1.30	1.04
PITRM1	pitrilysin metallopeptidase 1	1.13	4.09	1.58	0.00
PNN	pinin, desmosome associated protein	1.45	4.09	1.20	0.00
PNO1	partner of NOB1 homolog (S. cerevisiae)	1.10	4.09	1.31	0.00
PPAP2B	phosphatidic acid phosphatase type 2B	1.15	4.09	1.50	0.00
PPME1	protein phosphatase methylesterase 1	0.82	0.00	0.71	2.90
PPP1R13L	protein phosphatase 1, regulatory subunit 13 like	0.84	0.00	0.84	3.72
PPP1R3C	protein phosphatase 1, regulatory subunit 3C	1.40	4.09	5.72	0.00
PPP2CA	protein phosphatase 2, catalytic subunit, alpha isozyme	1.17	4.09	1.28	0.00
PPP2R1B	protein phosphatase 2, regulatory subunit A, beta	1.23	4.09	1.28	1.04
PPP3CA	protein phosphatase 3, catalytic subunit, alpha isozyme	1.18	4.09	1.26	0.00
PPP3CB	protein phosphatase 3, catalytic subunit, beta isozyme	0.86	0.00	0.42	2.90
PPP6R3	protein phosphatase 6, regulatory subunit 3	1.18	4.09	1.17	1.04
PRCC	papillary renal cell carcinoma (translocation-associated)	1.19	4.09	1.55	1.04
PREPL	prolyl endopeptidase-like	0.84	0.00	0.81	3.61
PRKAR2A	protein kinase, cAMP-dependent, regulatory, type II, alpha	1.18	4.09	1.45	0.00
PRNP	prion protein	0.85	0.00	0.82	3.72
PROSER1	proline and serine rich 1	1.17	4.09	1.40	0.00
PRPF39	PRP39 pre-mRNA processing factor 39 homolog (S. cerevisiae)	1.28	4.09	1.21	1.04

Gene symbol	Gene title	GSE13449 Fold change	GSE13449 FDR (%)	GSE34512 Fold change	GSE34512 FDR (%)
PRPF40A	PRP40 pre-mRNA processing factor 40 homolog A (S. cerevisiae)	1.31	4.09	1.31	0.00
PRPF4B	PRP4 pre-mRNA processing factor 4 homolog B (yeast)	1.32	4.09	1.47	0.00
PRR11	proline rich 11	1.31	4.09	2.43	0.00
PRRC1	proline-rich coiled-coil 1	1.17	4.09	1.14	1.04
PRRC2A	proline-rich coiled-coil 2A	1.22	4.09	1.68	0.00
PSMA1	proteasome (prosome, macropain) subunit, alpha type, 1	1.11	4.09	1.16	0.00
PSMB2	proteasome (prosome, macropain) subunit, beta type, 2	1.14	4.09	1.40	0.00
PSMC1	proteasome (prosome, macropain) 26S subunit, ATPase, 1	1.10	4.09	1.31	0.00
PSMD11	proteasome (prosome, macropain) 26S subunit, non-ATPase, 11	1.17	4.09	1.28	0.00
PSMD12	proteasome (prosome, macropain) 26S subunit, non-ATPase, 12	1.12	4.09	1.54	0.00
PUM2	pumilio homolog 2 (Drosophila)	1.15	4.09	1.18	1.04
QKI	QKI, KH domain containing, RNA binding	1.22	4.09	1.44	0.00
RAB11FIP1	RAB11 family interacting protein 1 (class I)	1.13	4.09	1.30	0.00
RAB21	RAB21, member RAS oncogene family	1.14	4.09	1.37	0.00
RABEP1	rabaptin, RAB GTPase binding effector protein 1	1.15	4.09	1.21	0.00
RABL3	RAB, member of RAS oncogene family-like 3	1.20	4.09	1.55	0.00
RAD23B	RAD23 homolog B (S. cerevisiae)	1.15	4.09	1.21	1.04
RBBP6	retinoblastoma binding protein 6	1.87	4.09	3.86	0.00
RBBP8	retinoblastoma binding protein 8	0.88	0.00	0.84	2.90
RBM25	RNA binding motif protein 25	1.33	4.09	1.36	0.00
RBM39	RNA binding motif protein 39	1.18	4.09	1.26	0.00
RBM4	RNA binding motif protein 4	0.87	0.00	0.79	2.90
RBMS1	RNA binding motif, single stranded interacting protein 1	1.15	4.09	1.68	0.00
RBPJ	recombination signal binding protein for immunoglobulin kappa J region	1.25	4.09	2.32	0.00
RCN1	reticulocalbin 1, EF-hand calcium binding domain	1.16	4.09	1.22	0.00
RFX7	regulatory factor X, 7	1.43	4.09	1.74	0.00
RHEB	Ras homolog enriched in brain	1.20	4.09	1.51	0.00
RING1	ring finger protein 1	1.14	4.09	1.34	0.00
RLN2	relaxin 2	0.78	0.00	0.56	2.90
RND3	Rho family GTPase 3	1.69	4.09	1.45	0.00
RNF6	ring finger protein (C3H2C3 type) 6	1.29	4.09	1.37	0.00
RRAGC	Ras-related GTP binding C	1.14	4.09	1.37	0.00
RRAS2	related RAS viral (r-ras) oncogene homolog 2	0.86	4.09	0.88	3.72
RREB1	ras responsive element binding protein 1	1.12	4.09	1.52	0.00
RSL1D1	ribosomal L1 domain containing 1	1.13	4.09	1.40	0.00
RSRC2	arginine/serine-rich coiled-coil 2	1.23	4.09	1.42	0.00
RUFY1	RUN and FYVE domain containing 1	1.14	4.09	1.45	0.00
RUFY3	RUN and FYVE domain containing 3	1.42	4.09	1.86	0.00
S100A10	S100 calcium binding protein A10	1.12	4.09	1.36	3.24
SAFB	scaffold attachment factor B	1.11	4.09	2.16	0.00
SCAF4	SR-related CTD-associated factor 4	1.12	4.09	1.11	0.00
SCARB2	scavenger receptor class B, member 2	1.20	4.09	1.83	0.00
SDC2	syndecan 2	0.87	0.00	0.64	2.90

Gene symbol	Gene title	GSE13449 Fold change	GSE13449 FDR (%)	GSE34512 Fold change	GSE34512 FDR (%)
SEC14L1	SEC14-like 1 (S. cerevisiae)	1.21	4.09	1.74	0.00
SEC24D	SEC24 family, member D (S. cerevisiae)	0.81	0.00	0.67	3.72
SEC61G	Sec61 gamma subunit	1.16	4.09	1.68	0.00
SEMA4C	sema domain, immunoglobulin domain (Ig), transmembrane domain (TM) and short cytoplasmic domain, (semaphorin) 4C	1.24	4.09	1.86	0.00
SENP6	SUMO1/sentrin specific peptidase 6	1.18	4.09	1.21	0.00
SEP15	15 kDa selenoprotein	1.15	4.09	1.31	0.00
SEPT7	septin 7	1.15	4.09	1.17	0.00
SEPT8	septin 8	0.85	0.00	0.77	2.90
SERINC3	serine incorporator 3	1.27	4.09	1.18	0.00
SET	SET nuclear oncogene	1.21	4.09	1.32	0.00
SETD1B	SET domain containing 1B	1.12	4.09	1.27	0.00
SETD5	SET domain containing 5	1.16	4.09	1.43	0.00
SFPQ	splicing factor proline/glutamine-rich	1.15	4.09	1.77	0.00
SGPL1	sphingosine-1-phosphate lyase 1	0.79	0.00	0.34	2.90
SGPP1	sphingosine-1-phosphate phosphatase 1	1.12	4.09	8.13	0.00
SH3GLB2	SH3-domain GRB2-like endophilin B2	1.21	4.09	1.58	1.04
SH3YL1	SH3 domain containing, Ysc84-like 1 (S. cerevisiae)	0.87	0.00	0.85	3.61
SIRT1	sirtuin 1	1.37	4.09	1.36	0.00
SKIL	SKI-like oncogene	1.11	4.09	1.54	0.00
SLC22A4	solute carrier family 22 (organic cation/ergothioneine transporter), member 4	1.29	4.09	1.38	0.00
SLC25A36	solute carrier family 25 (pyrimidine nucleotide carrier), member 36	1.33	4.09	1.40	1.04
SLC25A40	solute carrier family 25, member 40	0.89	0.00	0.50	2.90
SLC26A2	solute carrier family 26 (sulfate transporter), member 2	1.49	4.09	5.70	0.00
SLC2A10	solute carrier family 2 (facilitated glucose transporter), member 10	1.35	4.09	1.41	0.00
SLC31A1	solute carrier family 31 (copper transporters), member 1	1.17	4.09	1.16	3.24
SLC38A2	solute carrier family 38, member 2	1.21	4.09	1.43	0.00
SLC7A1	solute carrier family 7 (cationic amino acid transporter, y+ system), member 1	0.80	0.00	0.64	2.90
SLMO2	slowmo homolog 2 (Drosophila)	1.14	4.09	1.71	0.00
SMARCA4	SWI/SNF related, matrix associated, actin dependent regulator of chromatin, subfamily a, member 4	1.12	4.09	1.64	0.00
SMEK1	SMEK homolog 1, suppressor of mek1 (Dictyostelium)	1.31	4.09	1.72	0.00
SNCG	synuclein, gamma (breast cancer-specific protein 1)	2.34	4.09	1.94	1.04
SNRPA1	small nuclear ribonucleoprotein polypeptide A'	1.16	4.09	1.25	0.00
SNTB2	syntrophin, beta 2 (dystrophin-associated protein A1, 59kDa, basic component 2)	1.28	4.09	1.35	0.00
SNX2	sorting nexin 2	1.15	4.09	1.70	0.00
SORD	sorbitol dehydrogenase	1.10	4.09	2.85	0.00
SOWAHC	sosondowah ankyrin repeat domain family member C	1.29	4.09	1.18	0.00
SPG20	spastic paraplegia 20 (Troyer syndrome)	1.17	4.09	1.20	0.00
SREK1	splicing regulatory glutamine/lysine-rich protein 1	1.46	4.09	1.34	0.00
SRGAP2	SLIT-ROBO Rho GTPase activating protein 2	1.20	4.09	1.52	0.00
SRM	spermidine synthase	1.13	4.09	1.69	1.04
SS18	synovial sarcoma translocation, chromosome 18	1.26	4.09	2.08	0.00

Gene symbol	Gene title	GSE13449 Fold change	GSE13449 FDR (%)	GSE34512 Fold change	GSE34512 FDR (%)
SSH1	slingshot homolog 1 (Drosophila)	0.81	0.00	0.69	2.90
ST13	suppression of tumorigenicity 13 (colon carcinoma) (Hsp70 interacting protein)	1.11	4.09	1.42	0.00
STAMBP	STAM binding protein	0.82	0.00	0.74	3.61
STAU2	staufen, RNA binding protein, homolog 2 (Drosophila)	0.74	0.00	0.69	2.90
STC2	stanniocalcin 2	1.16	4.09	1.18	0.00
STEAP3	STEAP family member 3, metalloreductase	1.32	4.09	2.04	0.00
STRN3	striatin, calmodulin binding protein 3	1.19	4.09	1.79	0.00
STX3	syntaxin 3	1.29	4.09	2.37	0.00
SYNCRIP	synaptotagmin binding, cytoplasmic RNA interacting protein	1.12	4.09	1.39	0.00
TAB2	TGF-beta activated kinase 1/MAP3K7 binding protein 2	1.27	4.09	1.32	1.04
TAF1B	TATA box binding protein (TBP)-associated factor, RNA polymerase I, B, 63kDa	0.88	4.09	0.85	2.90
TAPBP	TAP binding protein (tapasin)	1.11	4.09	2.19	0.00
TBC1D2	TBC1 domain family, member 2	0.87	0.00	0.88	3.72
TBL1XR1	transducin (beta)-like 1 X-linked receptor 1	1.45	4.09	1.51	0.00
TCERG1	transcription elongation regulator 1	1.62	4.09	1.55	0.00
TCF3	transcription factor 3 (E2A immunoglobulin enhancer binding factors E12/E47)	1.29	4.09	1.29	0.00
TCF7L2	transcription factor 7-like 2 (T-cell specific, HMG-box)	0.82	0.00	0.87	2.90
TFAM	transcription factor A, mitochondrial	1.31	4.09	1.89	0.00
TFPI	tissue factor pathway inhibitor (lipoprotein-associated coagulation inhibitor)	3.89	4.09	1.75	0.00
TGFB2	transforming growth factor, beta 2	0.19	0.00	0.52	2.90
TGFBR2	transforming growth factor, beta receptor II (70/80kDa)	1.14	4.09	1.34	0.00
THUMPD1	THUMP domain containing 1	1.18	4.09	1.26	0.00
TJP1	tight junction protein 1 (zona occludens 1)	1.30	4.09	1.30	0.00
TMCC1	transmembrane and coiled-coil domain family 1	1.16	4.09	1.50	0.00
TMED2	transmembrane emp24 domain trafficking protein 2	1.15	4.09	1.51	0.00
TMED5	transmembrane emp24 protein transport domain containing 5	1.21	4.09	1.48	0.00
TMF1	TATA element modulatory factor 1	1.32	4.09	1.35	0.00
TMOD3	tropomodulin 3 (ubiquitous)	2.29	4.09	1.25	1.04
TNFRSF10B	tumor necrosis factor receptor superfamily, member 10b	1.15	4.09	3.01	0.00
TNPO1	transportin 1	1.11	4.09	1.19	1.04
TOB2	transducer of ERBB2, 2	1.72	4.09	1.93	0.00
TOP1	topoisomerase (DNA) I	1.27	4.09	1.13	1.04
TPM4	tropomyosin 4	1.45	4.09	1.67	0.00
TRAF3IP2	TRAF3 interacting protein 2	1.14	4.09	1.23	1.04
TRAF5	TNF receptor-associated factor 5	1.36	4.09	1.21	0.00
TRAK1	trafficking protein, kinesin binding 1	0.65	0.00	0.72	2.90
TRAM2	translocation associated membrane protein 2	1.19	4.09	1.34	0.00
TRIM13	tripartite motif containing 13	0.86	0.00	0.66	2.90
TRIM33	tripartite motif containing 33	1.15	4.09	1.14	0.00
TRMT1L	tRNA methyltransferase 1 homolog (S. cerevisiae)-like	1.40	4.09	3.30	0.00
TROVE2	TROVE domain family, member 2	1.70	4.09	1.20	0.00
TSN	translin	0.88	0.00	0.80	3.72
TSPAN4	tetraspanin 4	1.17	4.09	2.64	0.00

Gene symbol	Gene title	GSE13449 Fold change	GSE13449 FDR (%)	GSE34512 Fold change	GSE34512 FDR (%)
TTC37	tetratricopeptide repeat domain 37	1.20	4.09	1.28	0.00
TTF1	transcription termination factor, RNA polymerase I	1.51	4.09	1.21	0.00
TUSC2	tumor suppressor candidate 2	1.98	4.09	1.94	0.00
U2SURP	U2 snRNP-associated SURP domain containing	1.33	4.09	1.62	0.00
UBA2	ubiquitin-like modifier activating enzyme 2	0.88	0.00	0.83	3.61
UBE2H	ubiquitin-conjugating enzyme E2H	1.19	4.09	1.28	0.00
UBE2K	ubiquitin-conjugating enzyme E2K	1.11	4.09	1.11	0.00
UBE3A	ubiquitin protein ligase E3A	1.19	4.09	1.25	0.00
USO1	USO1 vesicle docking protein homolog (yeast)	1.29	4.09	1.14	0.00
USP32	ubiquitin specific peptidase 32	1.21	4.09	1.50	0.00
USP48	ubiquitin specific peptidase 48	1.20	4.09	1.37	0.00
VAMP4	vesicle-associated membrane protein 4	0.72	0.00	0.78	3.72
VCP	valosin containing protein	1.15	4.09	1.65	0.00
VEGFC	vascular endothelial growth factor C	0.90	0.00	0.71	2.90
WDR26	WD repeat domain 26	1.16	4.09	1.71	0.00
WHSC1	Wolf-Hirschhorn syndrome candidate 1	1.15	4.09	1.43	0.00
WRB	tryptophan rich basic protein	0.88	0.00	0.79	2.90
YAP1	Yes-associated protein 1	1.24	4.09	2.09	0.00
YTHDF3	YTH domain family, member 3	1.10	4.09	3.79	0.00
ZC3H15	zinc finger CCCH-type containing 15	1.24	4.09	1.17	1.04
ZC3HAV1	zinc finger CCCH-type, antiviral 1	1.27	4.09	1.55	0.00
ZFAND6	zinc finger, AN1-type domain 6	1.11	4.09	1.32	0.00
ZFHX3	zinc finger homeobox 3	1.60	4.09	1.73	0.00
ZFP36L1	zinc finger protein 36, C3H type-like 1	0.88	0.00	0.65	3.72
ZMIZ2	zinc finger, MIZ-type containing 2	0.85	0.00	0.87	3.72
ZNF12	zinc finger protein 12	1.21	4.09	1.49	0.00
ZNF136	zinc finger protein 136	1.20	4.09	1.57	0.00
ZNF160	zinc finger protein 160	1.21	4.09	1.34	0.00
ZNF174	zinc finger protein 174	1.16	4.09	4.31	0.00
ZNF238	zinc finger protein 238	0.71	0.00	0.75	2.90
ZNF329	zinc finger protein 329	1.26	4.09	1.79	0.00
ZNF432	zinc finger protein 432	1.24	4.09	1.36	0.00
ZNF467	zinc finger protein 467	1.45	4.09	1.15	1.04
ZNF84	zinc finger protein 84	1.32	4.09	1.24	0.00
